# Many Vulnerable or a Few Resilient Specimens? Finding the Optimal for Reintroduction/Restocking Programs

**DOI:** 10.1371/journal.pone.0138501

**Published:** 2015-09-22

**Authors:** María del Mar Gil, Miquel Palmer, Amalia Grau, Salvador Balle

**Affiliations:** 1 Laboratori d’Investigacions Marines i Aqüicultura, LIMIA (Balearic Government), Port d’Andratx, Balearic Islands, Spain; 2 Instituto Mediterráneo de Estudios Avanzados, IMEDEA (CSIC-UIB), Esporles, Balearic Islands, Spain; The Ohio State University, UNITED STATES

## Abstract

Most reintroduction and restocking programs consist of releasing captive-raised juveniles. The usefulness of these programs has been questioned, and therefore, quality control is advisable. However, evaluating restocking effectiveness is challenging because mortality estimation is required. Most methods for estimating mortality are based on tag recovery. In the case of fish, juveniles are tagged before release, and fishermen typically recover tags when fish are captured. The statistical models currently available for analyzing these data assume either constant mortality rates, fixed tag non-reporting rates, or both. Here, instead, we proposed a method that considers the mortality rate variability as a function of age/size of the released juveniles. Furthermore, the proposed method can disentangle natural from fishing mortality, analyzing the temporal distribution of the captures reported by fishermen from multiple release events. This method is demonstrated with a restocking program of a top-predator marine fish, the meagre (*Argyrosomus regius*), in the Balearic Islands. The estimated natural mortality just after release was very high for young fish (*m*_0_ = 0.126 day^-1^ for fish 180 days old), but it was close to zero for large/old fish. These large/old fish were more resilient to wild conditions, although a long time was needed to achieve a relevant reduction in natural mortality. Conversely, these large/old fish were more vulnerable to fishing, creating a trade-off in survival. The release age that maximizes the number of survivors after, for example, one year at liberty was estimated to be 1,173 days. However, the production cost of relatively old fish is high, and only a few fish can be produced and released within a realistic budget. Therefore, in the case of the meagre, increasing the number of released fish will have no or scarce effects on restocking success. Conversely, it is advisable implement measures to reduce the high natural mortality of young juveniles and/or the length of time needed to improve fish resilience.

## Introduction

The world is losing species and habitats at an alarming rate [1, 2], and various restoration efforts have been made to enhance or sustain local biodiversity. One commonly used conservation tool for many taxa, including mammals, birds, fish and reptiles, is to release captive-raised specimens into the wild through reintroduction and restocking programs [3]. These programs are usually based on releasing juveniles because, although subject to higher mortality than adults, they are assumed to be less affected by captivity and more readily adaptable to the wild [4, 5]. Moreover, producing juveniles is economically more efficient [6, 7].

Restocking and reintroduction projects have increased over the last 30 years, despite some skepticism on their effectiveness [8, 9]. Restocking has been criticized as ineffective and not economically viable. The limited usefulness of restocking has been attributed to the fact that cultured specimens may have lower survival, growth, reproductive fitness and genetic diversity than wild specimens [10, 11]. However, in some cases, releasing captive-raised animals represents the only management action available to reverse the negative trend of local biodiversity losses [12, 13]. Nevertheless, restocking programs are not usually quantitatively assessed [14]. To improve management, their success should be experimentally evaluated [15, 16].

A restocking program can be considered successful when the released specimens survive to adulthood in sufficient numbers to rebuild a population with a stable age structure and to ensure the next generations of broodstock [17]. Thus, one of the final objectives of a restocking program is usually to re-establish a self-sustaining population. However, in the shorter term, one way to check the potential success of a restocking program is to ensure that at least some of the captive-raised and released specimens are able to survive until achieving sexual maturity and becoming potentially capable of reproduction in the wild. Therefore, the post-release survival of juveniles is a crucial parameter for assessing success.

Marking (tagging) experiments are one of the primary tools for studying mortality rate, and an extensive literature exists documenting the wide variety of experimental designs and estimation models available [18–20]. Marking experiments can be divided into those where marked individuals are recaptured and released multiple times and those where individuals die upon recapture so that only a single recapture event is possible [21]. In the first type, several recapture events provide a history of recaptures for each individual, which can allow researchers to estimate a number of the parameters for predicting population dynamics. A rich statistical literature has been developed for these experiments [20, 22], which have been applied to a wide range of non-exploited mammals, reptiles and birds [23]. In the second type of marking experiment, animals (typically, fish and birds) are part of an exploited population and are recovered dead upon harvest. Polacheck, *et al*. [21] divided these single-recapture experiments into two fundamentals types: the tag-attrition model (a single release event) and the Brownie model [24], with multiple release events. The tag-attrition model provides estimates of mortality from the rate at which tags are reported over time [25]. However, natural mortality needs to be assumed known, or both fishing and natural mortality rates need to be assumed constant [21, 26]. The Brownie model provides estimates of total mortality rates by comparing the reporting rates over time from multiple releases [27]. In the Brownie model, annual cohorts are tagged in different years and the tags are collected over a period of years [18]. A single (usually annual) estimate of mortality is provided for the period between any two tagging events. However, the usefulness of both types of models may be severely limited by unreported tags (i.e., some animals are captured but these captures remain unknown). The reporting rate needs to be known or independently estimated when applying the tag-attrition model. In the case of the Brownie approach, an estimate of total mortality can be obtained if the unknown reporting rate is assumed constant over time, but the precision of this estimate is usually unsatisfactory [28]. Therefore, the methods available are limited by the need to know the non-reporting rate and/or the problems related with mortality that varies through time.

Nevertheless, adapting to natural habitats and natural food sources and avoiding predation is critical for the survival of captive-raised and released specimens. Post-release survival can be negatively affected by the inexperience of released animals to face the new environment [9, 29]. For example, in the Barn owl (*Tyto alba*), starvation is one of the most important causes of death in released captive-reared animals [30]. In turbot (*Psetta maxima* and *Scophthalmus maximus*), poorly developed burying ability and escape response from predators increase mortality [31, 32]. In fact, a common feature of restocking projects seems to be a high loss of animals immediately after release [33]. The elevated mortality just after release seems to improve over time, and therefore, minimizing the immediate mortality and the length of the period during which post-release mortality rates are high could be effective for boosting survival rates [32]. Given the general agreement that mortality is not constant, we propose a new model that explicitly considers mortality as age/size dependent. We evaluate our model on the effectiveness of the restocking program of a top-predator marine fish, the meagre *Argyrosomus regius* (Asso, 1801), that is currently being conducted at the Balearic Islands, Western Mediterranean.

The meagre is a large sciaenid that is widely distributed along the eastern Atlantic coast (from Norway to Congo) and the whole Mediterranean [34]. This species is highly vulnerable to both overfishing and environmental degradation of spawning habitats, and its abundance has decreased alarmingly in the Mediterranean [35, 36]. It is considered to be extinct in the Balearic Islands, where it was a relatively frequent capture only a few decades ago [37]. Therefore, the Balearic Government qualified meagre as a suitable species for a restocking program based on releasing hatchery-reared fish. Accordingly, more than 10,000 tagged juveniles have been released since 2008 and along multiple release events. The only source of information available on those fish after releasing is that some of them have been recaptured and reported by fishermen [38].

The ultimate goal of the approach proposed here is to assess the success of any restocking program for estimating the expected number of surviving animals after spending a target time at liberty. The data used for this purpose will be the distribution over time of the captures reported by fishermen. Moreover, we will demonstrate that this approach is able to disentangle natural from fishing mortality and how they change depending on fish age/size. This capability, in combination with knowledge of the production costs, may be used to find the optimal point between releasing a large number of small/young vulnerable individuals and a small number of large/old and more resilient fish [7].

## Methods

### Ethic statements

In relation to ethics concerns on the protocols of fish management used in this study, we declare:

1. The full (i.e. from rearing fish to releasing fish at the wild) restocking program of meagre has been approved by the ethics committee of LIMIA.
2. LIMIA is a governmental (Balearic Government) laboratory authorized for animal research (official register reference code ES070050000502). LIMIA staff has been officially qualified to rear, on-grow and label fish. These tasks were conducted under veterinary supervision and minimized fish suffering, which is mandatory for any Spanish registered laboratory for animal research. LIMIA certifies that rearing, growing and labeling fish was carried out in accordance with the recommendations of the Directive 2010/63/UE, relative to the protection of animals used for scientific purposes.
3. According to Spanish law (RD 53/2013, BOE 34/2013), the protocols used for identifying an animal (labelling) are not considered experimental practices and, thus, do not require prior authorization (art. 2.5 of the aforementioned RD). Internal tagging (bathing; see details below) was performed in tanks at low density and strong aeration to minimize stress. Prior to inserting external tags, fish were anesthetized (tricaine methanesulfonate or MS-222) to minimize suffering.
4. Similarly, no prior authorization for conducting experimental practices is needed when standard aquaculture protocols are used for rearing and on-growing fish. Reproduction and rearing processes were conducted following the production protocol developed at LIMIA [39] which specifies proper handling and production conditions to ensure the welfare of the fish. Meagre eggs were obtained at LIMIA by hormonal induction (Ovaprim hormone, Syndel Inc., Canada) of the broodstock maintained at LIMIA.
5. No fish were sacrificed for conducting this study.

In relation to ethics concerns on the protocols for promoting fisher implication in the restocking program, an ethics committee has not been consulted. Nevertheless, protocol design was done under an extreme precautionary approach consisting in:

1. Fishers were asked to freely collaborate to the meagre recovery program. Fishers were informed (leaflets) on the aim of the recovery program and on the nature and relevance of the collaboration.
2. Fishers might collaborate in two ways. First, freely answering a short and anonymous (no data on subject identification were kept) interview of only three very general questions (“Dou you know the recovery program of meagre?” “If yes, how did you know?” and “Have you ever fished a meagre?”). The objective was to check if the recovery program was known (outreach purposes). Second, fishers might collaborate reporting or communicating the capture date and location of marked fish. In that case, fishers received a small gift and voluntarily enter in a draw of fishing material. Fishers were informed in advance (leaflets) on the details of the small gifts and the draw.
3. Specifically, fishers were informed that neither the gift nor the draw could be considered a profit but a compensation for freely collaborating with the meagre recovery program.
4. Any data that can be used for identifying subjects were anonymized using codes. Both, codes and subject data, were destroyed just after the draw was done.
5. An advertisement campaign on the recovery program was done, where in addition to the goals of the recovery program, the protocol for reporting fish or communicating captures was publicized.

### Production, tagging and release

Approximately 2,000 juveniles per year were produced at LIMIA from 2008 to 2013.

Prior to release, all of the meagre juveniles were measured (total length, *L*_T_), weighed, marked internally with an alizarin bath [39] to distinguish between cohorts, and marked externally with a T-bar tag (Floy® T-Bar Anchor FF-94 and FD-94 tags) imprinted with an individual identification code and a phone number. After marking, juveniles were released at sites on the coast of Mallorca Island [38]. The numbers and lengths of released specimens in 21 release events are detailed in Table 1. Between-event variability in length (and age) at release was noticeable (15.3 cm to 51.5 cm; Table 1).

**Table 1:** Number of released meagres during the restocking program with their age, length and number of identified recaptured meagre with their corresponding days at liberty (DAL).

Release	Relase data	Birth day	Age (months)	*L*_T_ (cm)	Num. released	Num. recaptured	DAL
#1	05/11/2008	16/05/2008	6	15.3 ± 1.6	700	0	-
#2	05/11/2008	06/05/2007	18	31.9 ± 2.0	208	8	15–16
#3	07/11/2008	16/05/2008	6	15.6 ± 1.7	443	1	18
#4	17/02/2009	16/05/2008	10	17.3 ± 2.2	2,805	3	9–336
#5	24/02/2009	06/05/2007	22	34.1 ± 2.5	1,272	121	1–1,560
#6	16/11/2009	16/05/2008	19	39.1 ± 2.1	170	5	119–677
#7	18/03/2010	22/05/2009	11	23.6 ± 2.1	1,009	3	1–11
#8	16/04/2010	22/05/2009	12	25.5 ± 2.3	1,018	41	3–8
#9	21/09/2010	22/05/2009	17	32.9 ± 2.6	574	47	2–303
#10	20/10/2010	22/05/2009	18	34.1 ± 2.6	224	15	1–37
#11	14/12/2010	22/05/2009	20	35.7 ± 2.8	396	3	6–338
#12	14/12/2010	22/05/2009	20	36.7 ± 2.5	397	39	1–27
#13	16/12/2010	16/05/2008	32	51.5 ± 2.7	156	22	20–254
#14	03/02/2011	22/05/2009	21	36.3 ± 2.7	397	33	2–186
#15	18/05/2011	22/05/2009	25	45.6 ± 3.4	157	18	1–128
#16	22/07/2011	22/05/2009	27	46.6 ± 4.0	126	28	1–70
#17	20/09/2011	21/05/2010	17	32.3 ± 3.6	866	19	2–639
#18	22/11/2012	19/05/2012	7	20.1 ± 2.0	1,014	1	1
#19	14/02/2013	19/05/2012	10	23.1 ± 2.3	510	1	2
#20	14/05/2013	19/05/2012	13	25.2 ± 1.8	288	3	32–61
#21	26/07/2013	19/05/2012	15	26.8 ± 2.7	404	2	1–49
**Total**					**13,134**	**413**	

### Recaptures

Although the term may cause some confusion, to be consistent with other marking studies, here we use the term recapture to describe fish that have been released and captured after spending some time at the wild. Meagres can be recaptured by commercial (with trammel nets) or recreational (with fishing rods or spear guns) fishermen along the entire Mallorca coast. Fishermen are the only source of information about the time when the released fish were recaptured, and we expect them to be motivated to reliably report recaptures because of the increasing public concern about ecological sustainability of fishing [40]. An advertising campaign was designed and implemented to inform the fishermen about the meagre restocking program and the steps to take if a meagre was caught. The communication program consisted of i) television, radio and press appearances, ii) poster and leaflet distribution, and iii) personal interviews. On several occasions, the scientists involved in the meagre restocking program appeared in local media to inform the population about the objectives of the project. Furthermore, just before each release event, informative posters were distributed to all of the fishing shops, diving communities and professional fishermen’s associations in Mallorca. Leaflets were primarily distributed at Palma Bay, an area with many recreational and commercial fishermen. Finally, face-to-face interviews were also performed to obtain data on the penetration of the advertisement campaign among the fishers and to obtain additional data on non-reported captures of meagre. These interviews were also opportunities to explain the details of the restocking program to the fishers and to instruct them about what to do when recapturing a meagre.

When a meagre was recaptured and the fishermen called us using the phone number printed on the T-bar tag, they provided information about the recapture date and location (as precisely as possible), approximate depth of fishing, type of gear and approximate length or weight. A gift was given to fishermen who reported recaptured meagres.

Furthermore, an automatic notification system was implemented, and information on any meagre sale in the fish auction of the central fish market of Mallorca Island was obtained almost instantly. We could therefore obtain information of some of the recaptured meagres that otherwise would have remained unreported or that had lost the external tag after a long period in the wild [41]. These long-term recaptures were very important for improving the precision and accuracy of mortality estimation. In some cases, these fish were located just before they were sold to the final customer. When possible, in these cases, fish were bought to verify the release data by aging the fish and observing the alizarin mark in the otolith. The release date was determined through the external tag, age or alizarin mark when the otolith was available or by the length/weight for a few very clear cases. The recapture date was recorded during an interview of fishermen. Recaptured meagres with imprecise or doubtful information were excluded from further analyses.

### Theoretical framework

Provided that the only source of information available after a releasing event are the fish that have been captured and reported by fishermen, the ultimate goal of the approach proposed here is to estimate the expected number of surviving fish at any time using the distribution over time of the reported captures, the number of fish released and the age/size at release. The number of reported fish at a given time depends on the number of captured fish, which in turn depends on the surviving fish at that time. The number of surviving fish will be the difference between the released fish and the accumulated number of dead fish from the release, but fish can die either by natural causes (e.g., starvation or predation) or by fishing. Moreover, the abovementioned empirical evidences suggest that natural and fishing mortality may depend on fish size/age, thus they will depend on the size/age at release and will change with fish growing at the wild.

Therefore, the estimation of mortality requires an underlying dynamic model of how the number of animal varies over time. In the restocking case, neither birth nor immigration are expected to occur; therefore, the variation in the number of fish *N* is given by [26]:

$$\frac{dN}{dt}=-m(t)N-f(t)N$$ (1)

where *m*(*t*) is the natural mortality and *f*(*t*) is the fishing mortality. The solution of this differential equation when *m* and *f* are constant in time is the well-known exponential model [42]

$$N(t)=N_{0}e^{-(m+f)t}$$ (2)

where *N*_*0*_ is the initial number of released fish. However, the assumption of constant mortality is unrealistic. For example, natural mortality of captive-raised released specimens is expected to decrease with age because they may require an adaptation period to the wild conditions [38]. Therefore, when either *m* or *f* are size/age dependents, the number of fish that survive at a time *t* after being released with age *t*_0_ is given by:

$$N(t,t_{0})=N_{0}(t_{0})e^{-\int_{t_{0}}^{t+t_{0}}[m(t')+f(t')]dt^{'}}=N_{0}(t_{0})e^{-M(t,t_{0})-Q(t,t_{0})}$$ (3)

where *M*(*t*,*t*_*0*_) refers to the part of the integral related with natural mortality and *Q*(*t*,*t*_*0*_) to the part related with fishing.

Concerning natural mortality, we propose that variation in the instantaneous natural mortality rate, *m*(*t*), could be expressed as:

m(t)=m∞+(m0−m∞)e−tτ (4)

which seems appropriate for describing the biological process of adaptation to the wild conditions: natural mortality is maximal when fish are released (*m*_*0*_) but *m*(*t*) decreases toward a smaller asymptotic mortality (*m*_*∞*_) as fish increase the size/age at the wild. Note that the parameter *τ* determines how fast the change in mortality occurs (the smaller *τ*, the faster is the change in mortality).

Solving *M*(*t*,*t*_*0*_):

M(t,t0)=∫t0t+t0[m∞+(m0−m∞)e−t'τ]dt'=m∞t+(m0−m∞)τe−t0τ(1−e−tτ) (5)

Concerning fishing mortality, we propose that the instantaneous mortality rate would be size-dependent in the sense that no fish are expected to be captured below a certain size, but thereafter, *f*(*L*) would be proportional to fish length:

$$f(L)=\alpha (L-L_{f})\theta (L-L_{f})$$ (6)

where *L* is the fish length, *L*_*f*_ is the threshold length from which a fish is vulnerable to fishing, and the *θ* function is defined as *θ(x)* = 0 when *x* < 0 and *θ(x)* = 1 when *x* > 0. Note that this is simply an appropriate way for describing the vulnerability of fish exploited by size-selective gear, which typically remove larger and therefore older individuals [43, 44]. Assuming that *L*(*t*) is described by the conventional von Bertalanffy growth model,

$$f(L)=f(t)=\alpha L_{\infty }(e^{-kt_{f}}-e^{-kt})\theta (t-t_{f})$$ (7)

where *k* and *L*_*∞*_ are parameters of the von Bertalanffy growth equation and *t*_*f*_ is the age corresponding to the length from which a fish is vulnerable (*L*_*f*_). Note that fishing effort was assumed temporary constant, therefore have no effect on fishing mortality.

Solving *Q*(*t*,*t*_*0*_):

$$\begin{array}{l} Q(t,t_{0})=\alpha L_{\infty }e^{-kt_{0}}\left[\left(t+t_{0}-t_{f})\theta (t+t_{0}-t_{f})-(t_{0}-t_{f})\theta (t_{0}-t_{f}\right)\right]-\\ \;-\alpha L_{\infty }\left[\theta (t+t_{0}-t_{f})\frac{e^{-kt_{f}}-e^{-k(t+t_{0})}}{k}+\theta (t_{0}-t_{f})\frac{e^{-kt_{0}}-e^{-kt_{f}}}{k}\right] \end{array}$$ (8)

Finally, the number of fish captured (*F*) at time *t* after release will be:

$$F(t,t_{0})=f(t)N_{0}(t_{0})e^{-M(t,t_{0})-Q(t,t_{0})}$$ (9)

and the expected number of reported fish (*R*) will then be:

$$R(t,t_{0})=rf(t)N_{0}(t_{0})e^{-M(t,t_{0})-Q(t,t_{0})}$$ (10)

where *r* is the reporting rate, which is assumed to be constant.

### Model fitting

When τ >> Δ*t*, Eq 10 can be discretized (here Δ*t* was set to 10 days) as:

$$R_{t,t_{0}}=rf_{t}\Delta tN_{0}{}_{t0}e^{-M(t,t_{0})-Q(t,t_{0})}$$ (11)

where *R*_*t*,*t0*_ is the matrix of (columns) the number of meagre recaptured and reported for the commercial and recreational fishery from *t* = 1 to *t* = 1,600 days (to include all recaptures) and (rows) for each of the 21 release events considered.

The observed value $R_{t,t_{0}}^{obs}$(i.e., the number of fish reported during the *t* ^*th*^ time period corresponding to a release event of *N*_*0*_ fish and *t*_*0*_ days old) is assumed to be Poisson distributed with mean equal to $R_{t,t_{0}}$. Therefore, the likelihood is given by:

$$f(R_{t,t_{0}}^{obs}|R_{t,t_{0}})=\frac{R_{t,t_{0}}^{R_{t,t_{0}}^{obs}}e^{R_{t,t_{0}}}}{R_{t,t_{0}}^{obs}!|}$$ (12)

The six unknown parameters are *r* (tag reporting rate), *α* (slope of the relationship length *versus* fishing mortality), *t*_*f*_ (age from which a fish is vulnerable to fishing), *m*_*0*_, *m*_*∞*_ and *τ* (parameters of natural mortality; Eq 4). Conversely, note that *t*_*0*_ and *N*_*0*_ (the age and the number of released fish at each one of the releasing event) are known. The von Bertalanffy growth rate (*k*) and maximum length (*L*_*∞*_) have been independently estimated with the length-at-age data from all of the released fish (i.e., *k* = 6.37 10^−4^ days^-1^ and *L*_*∞*_ = 115.2 cm).

The age from which a fish is vulnerable (*t*_*f*_) was set to 200 days old (13.8 cm) because no fish younger were captured (see below). The other five unknown parameters were estimated using a Bayesian approach. A weak gamma distribution (shape = 0.001, scale = 0.001) was assumed as priors for *m*_0_, *m*_∞_ and *α*. The tag reporting rate (*r*) and *τ* were constrained to be, respectively, within the intervals (0, 1) and (0, 1000 days). Three MCMC chains were run using randomly selected initial values for each parameter within a reasonable interval, and conventional convergence criteria were checked. The number of iterations was selected for each run to obtain at least 1,000 valid values per chain after convergence and thinning (1 out of 100). The model was implemented with the library *R2jags* (http://cran.r-project.org/web/packages/R2jags/R2jags.pdf) of the R-package (at http://www.r-project.org/) that uses the samplers implemented in JAGS (http://mcmc-jags.sourceforge.net/). The R script (Sub-file A in S1 File) and data (Sub-file B in S1 File) for reproducing the analysis are provided as supporting information.

### Simulation experiments

To demonstrate the outcomes of the results in terms of number of surviving fish, the point estimates of the parameters have been used to complete two simulation experiments. In the first simulation experiment, three sets of the same number of fish (10,000) of increasing age (500, 1,500 and 3,000 days old) were released, and the number of surviving fish after spending different times at liberty (from 1 to 1,000 days) was recorded. In the second simulation experiment, up to 100 sets of fish of increasing age (from 180 to 2,000 days old) were simulated, but in this case, the number of fish of each set is not fixed, but corresponds to the number of fish that can be produced with a given budget. The relationship between cost-per-fish (*CO*) and age/length takes into account feeding cost (*FCO*) and personal cost (*PCO*) [6]:

CO=FCO+PCO (13)

Where the feeding cost (*FCO*) depends on the price of the diet (€ kg^−1^) and the accumulated daily food ration (kg), which depends on the temperature, fish size and pellet size. The personnel cost per fish (*PCO*) depends on the salary per day (*S*), the number of fish finally produced in a given year and the days (*D*) required on each diet to achieve the desired length [6].

The simulated budget was set to 10,000 euros/year. In this simulation, the number of fish finally released was readjusted after taking into account fish losses within the cages. Specifically, a daily mortality rate of 0.012% per day^-1^ was assumed, based on empirical data from LIMIA (unpublished data). In addition to this, a limit capacity of 10,000 fish was established for the facilities. Furthermore, in the second simulation, the number of surviving fish after one year at liberty was recorded.

## Results

### Analysis of meagre collected data

**Fig 1:**
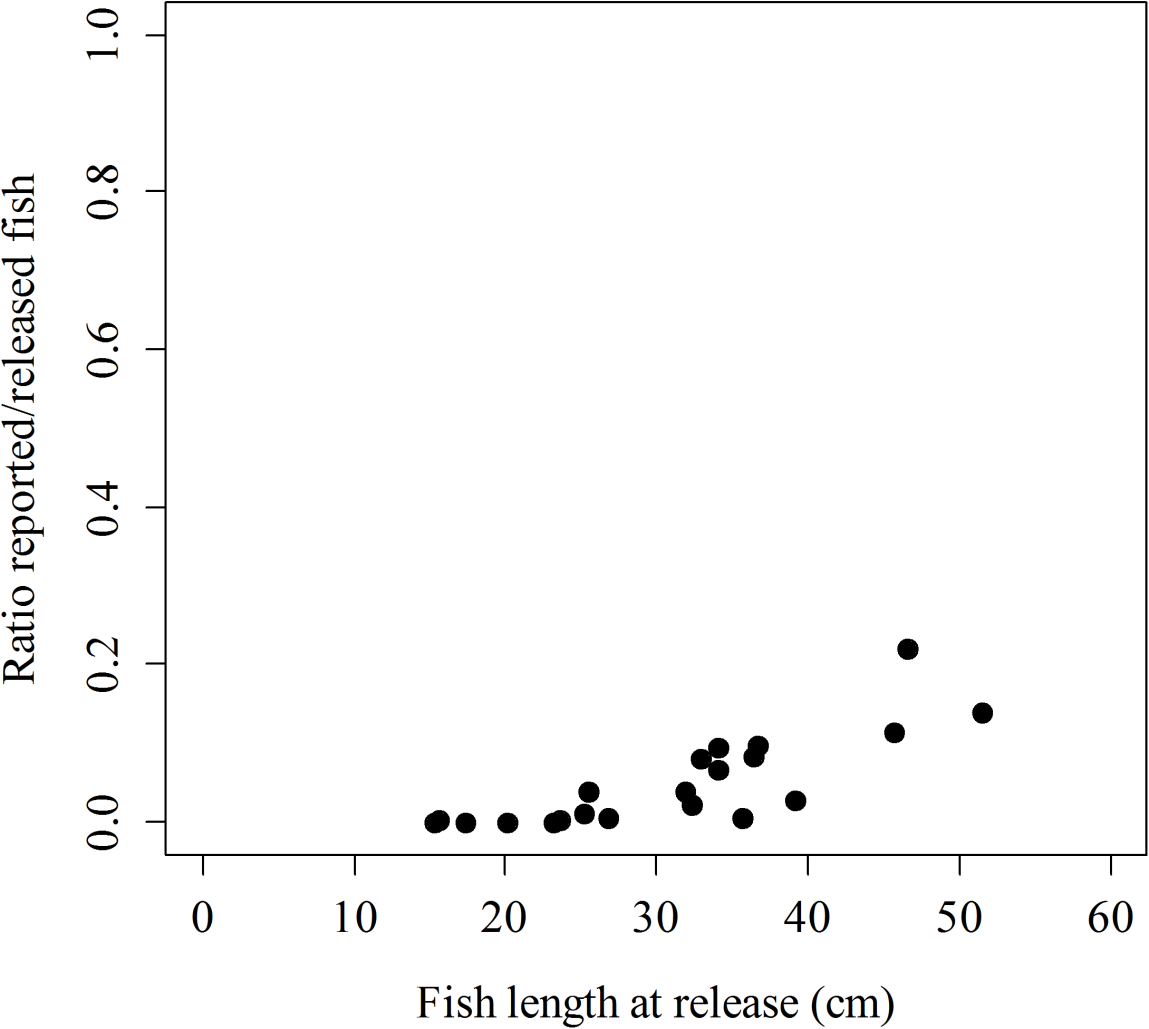
Relationship between fish length at release and the ratio of number of fish reported / number of fish released.

During the restocking program, a total of 13,134 meagres (Table 1) were released, and data from 429 recaptured meagres were recorded, although just 413 of these recaptures had accurate information about the release and recapture date. Therefore, the ratio of reported/released of this study was 3.3%, although this ratio ranged from 0–0.1% for release events of small fish to 14–23% for release events of large fish (Fig 1). Note that no fish smaller than 13.8 cm were reported and that the ratio of reported/released increased with the fish length at release (Fig 1). However, interpreting this ratio is not straightforward because high values can be the outcome of two confounding processes that demand a very different management strategy: either a reduction of the natural mortality or an increased vulnerability to fishing.

**Fig 2:**
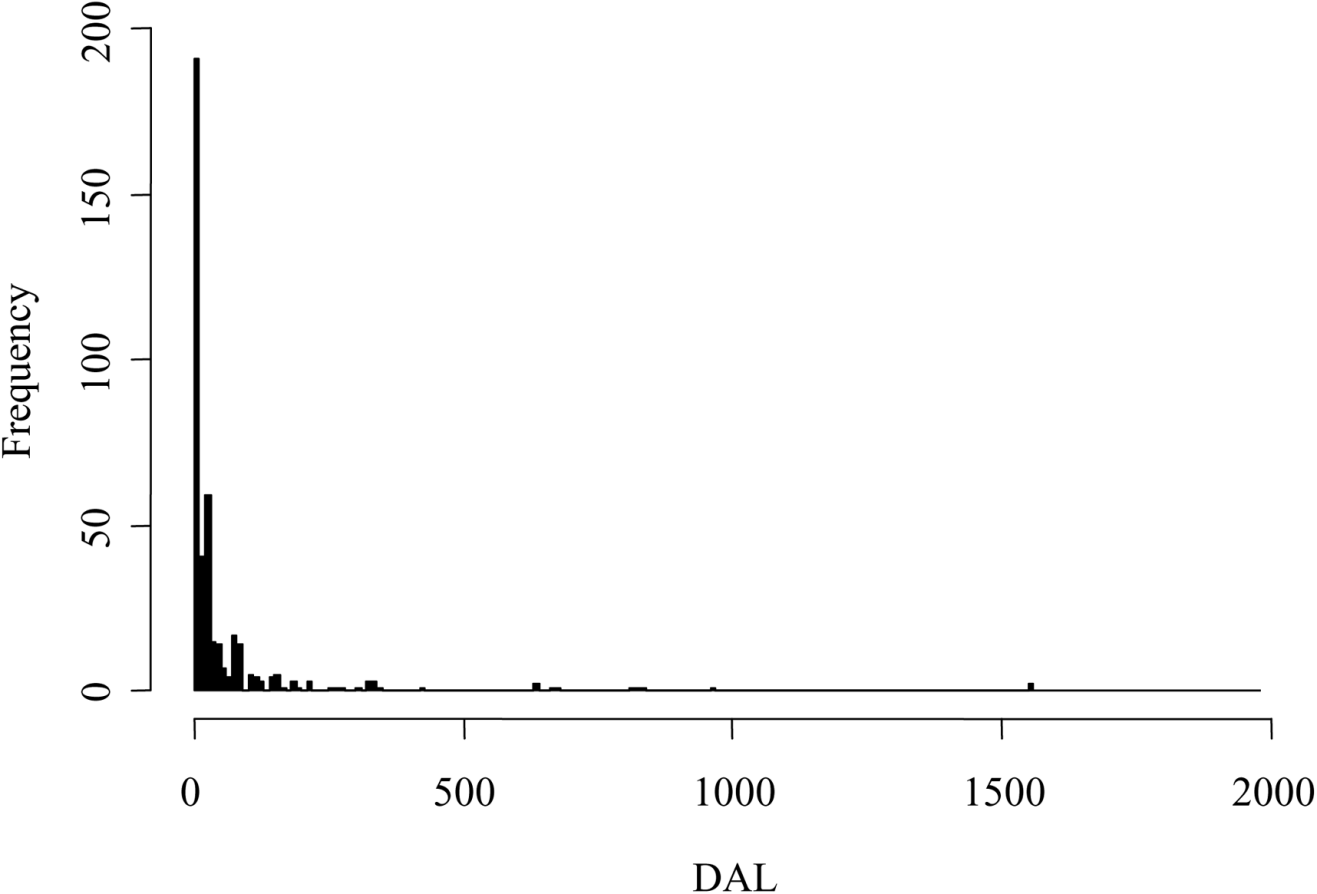
Frequency histogram of meagres reported over days at liberty (DAL). The width of the bars corresponds to 10 days at liberty.

The observed time at liberty ranged from 1 to 1,560 days after release. The distribution of recaptured meagres over time presented an exponential-like distribution (Fig 2), with most of the captures clustering just after release. However, it is noticeable that some fish were captured after more than four years at liberty; thus, the distribution of recaptures was clearly overdispersed. Assuming a constant fishing effort, this pattern strongly suggests that instantaneous mortality rates, both natural *m*(*t*) and fishing *f*(*t*) mortalities, are not constant over time.

**Fig 3:**
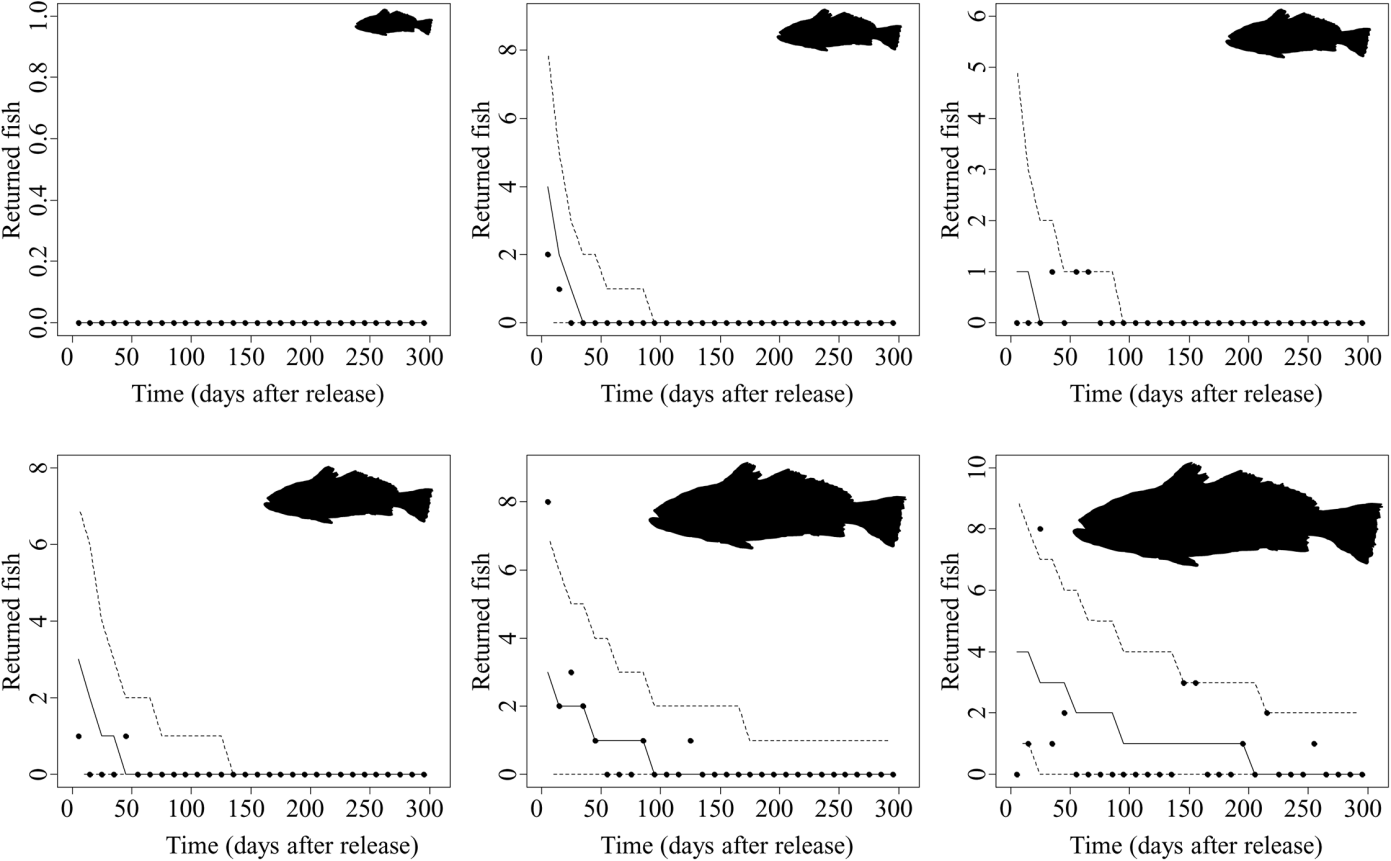
Comparing the predicted by the model and the actually observed number of reported fish (solid points) at any given 10-days period corresponding to six releasing events selected for having a progressively larger size-at-release. For a given release event, 3.000 predictions of the number of reported fish were estimated using each one of the 3.000 values from the posterior distributions of the model parameters. A random sample from a Poisson distribution was then obtained for setting the (95%) credibility intervals (dashed lines) and median values (solid line).

Both patterns (overdispersion and increasing the reported/released ratio with fish length at release) are well explained by the proposed model. The number of reported fish at any given 10-days period predicted by the model are compared with the actually observed number of reported fish in Fig 3. When releasing small fish, the number of reported fish was always zero or quickly decreased, because natural mortality was elevated and large/old fish (i.e., fish that have spent long time at liberty) were never reported. Conversely, when releasing large fish, the decrease was slow and some fish were reported during an extended period.

**Fig 4:**
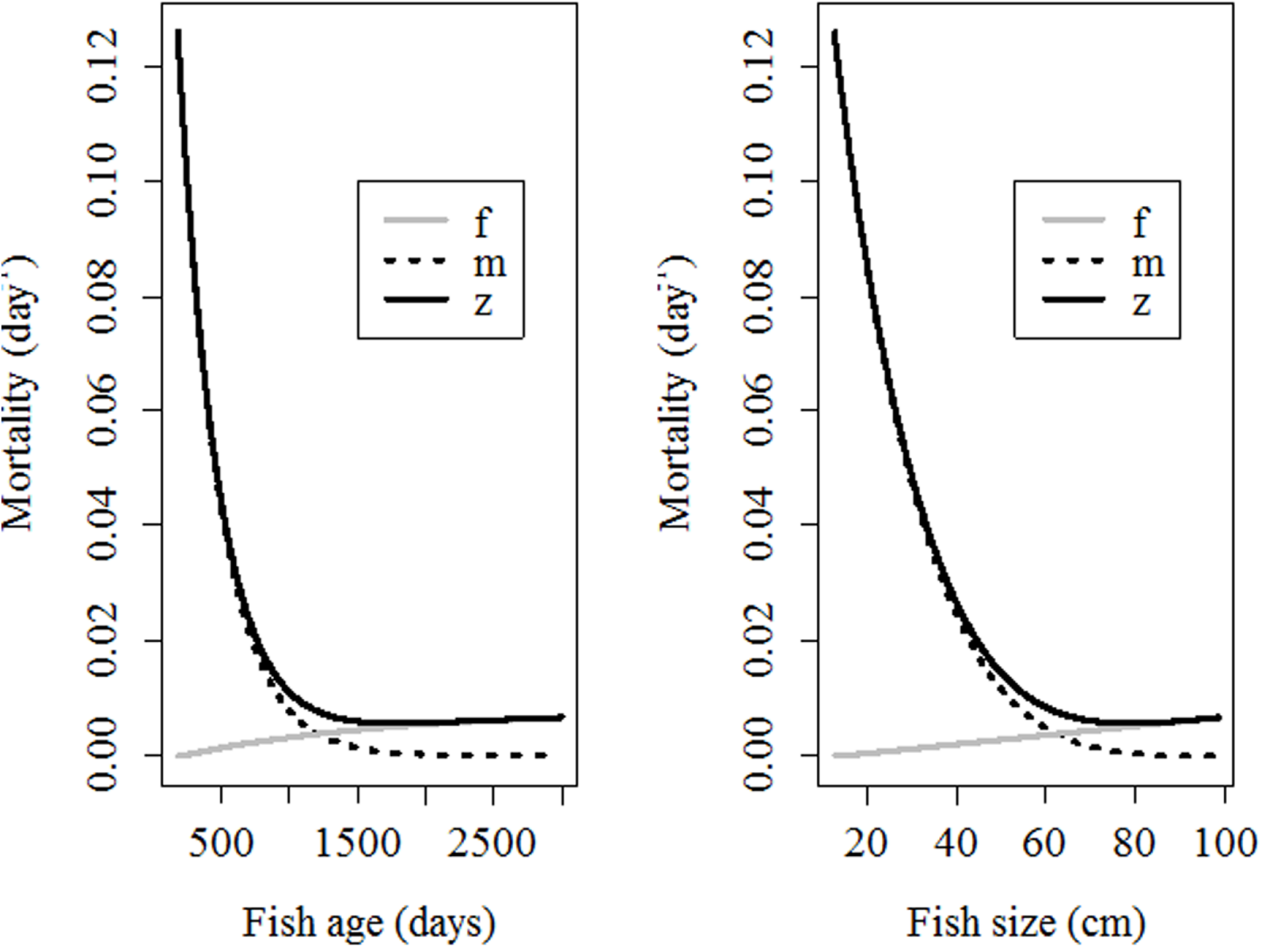
Expected changes in total mortality (*z*), natural mortality (*m*) and fishing mortality (*f*) in relation to fish age (left) and fish length (right). Note the high values of natural mortality for young fish and its sharp decrease. In contrast, fishing mortality increases at a low rate.

A reasonable convergence after Bayesian inference was achieved for most of the parameters of the model considered. The median and 95% percentiles of the Bayesian credibility intervals are shown in the Table 2. The estimated reporting rate (*r*) was surprisingly high, ranging (95% percentiles) from 0.72 to 0.99, which suggests that commercial and recreational fishermen are more concerned with the need for a sustainable management than previously suspected. Nevertheless, the credibility interval for *r* was relatively wide. In contrast, precision for the four mortality-related parameters was better, which supports the capability of the model for predicting and inferring patterns. Concerning natural mortality, note that the value of *m*_*0*_ was 0.231 days^-1^ at age = 0 days. The corresponding figure for a release age = 180 days (which corresponds with the minimum age of released meagres in this study) is 0.126 days^-1^, or 12.6% of fish dead each day. However, *m* drops to virtually zero for fish that spent a long time at liberty (*m*_*∞*_ *=* 10^−21^ days^-1^), but this decrease is slow (*τ* = 298 days). In contrast, fishing mortality increases with fish length but at a low rate (*α* = 7.9 10^−5^ day^-1^cm^-1^) and only for fish larger than 13.8 cm because smaller fish are assumed not to be vulnerable to fishing. The patterns estimated for natural and fishing mortality are displayed in Fig 4.

**Table 2:** Median and 95% credibility interval for initial natural mortality (*m*_0_), final natural mortality (*m*_∞_), rate of change between *m*_0_ and *m*_∞_ (*τ*), slope of the relationship between fishing mortality and fish length (*α*) and report rate (*r*).

Parameter	Lower 95% C.I.	Median	Upper 95% C.I.
*m*_0_ (day^-1^)	0.18	0.23	0.30
*m*_∞_ (day^-1^)	0	10^−21^	10^−4^
*τ* (day)	265	298	332
*α* (day^-1^cm^-1^)	6.6 10^−5^	7.9 10^−5^	9.9 10^−5^
*r*	0.72	0.92	0.99

The main applied interest of obtaining precise estimates of the mortality parameters is that the number of expected survivors at any given time after release can be estimated from the number of released fish and the size at release. The number of meagres that were expected to reach sexual maturity in the wild under the currently used setting (i.e., the 21 release events detailed in Table 1) was only 9.6 of the 13,134 released fish, and all of them came from the release #13, which corresponds to the release of larger fish.

### Simulation experiments

**Fig 5:**
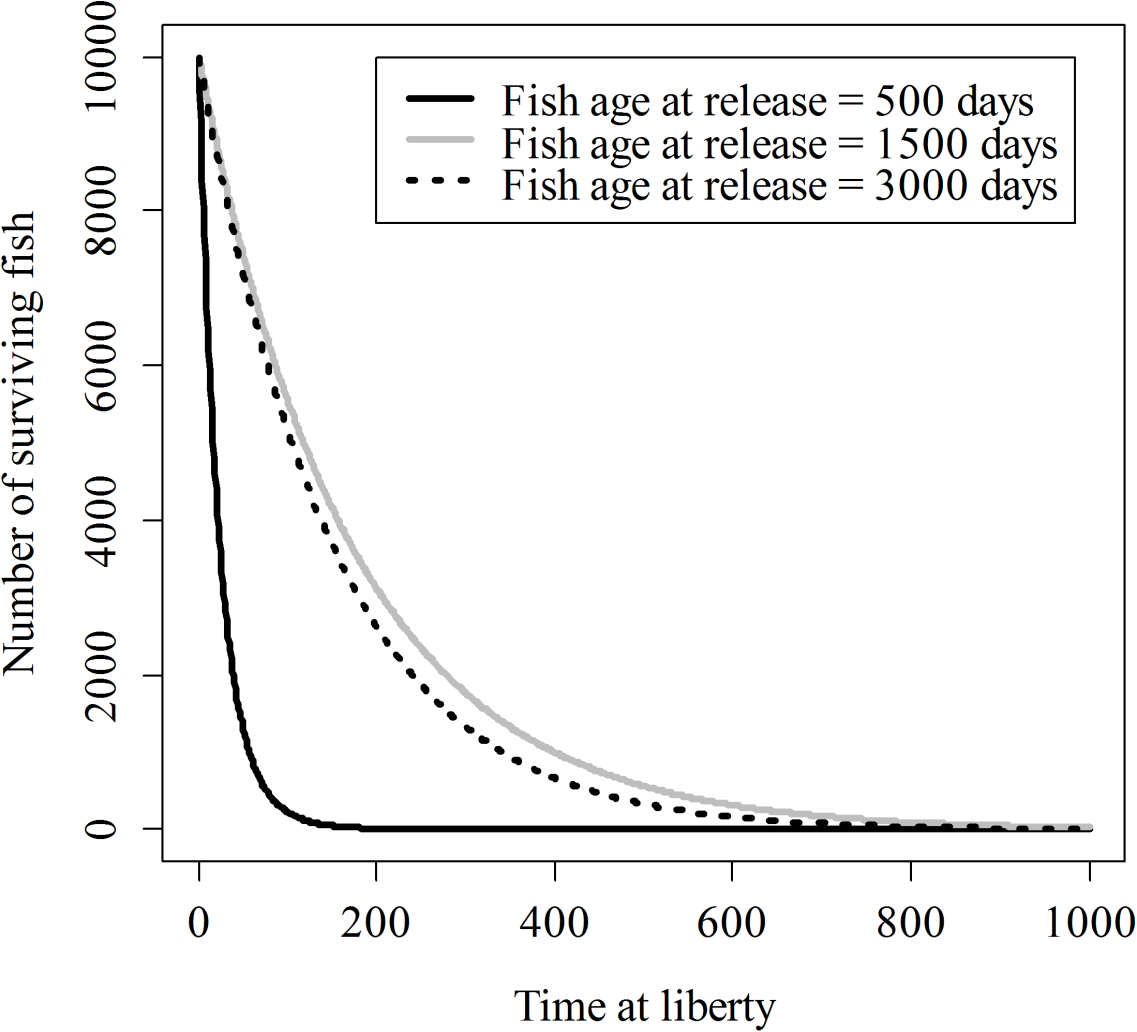
Expected number of surviving fish after releasing 10,000 fish. Note that in the three release events (500, 1,500 and 3,000 days old fish), fish of intermediate age seem to show better survivorship.

The relevance of the patterns displayed by natural and fishing mortality (Fig 4) is better visualized by the simulation experiments, which also uses the estimated parameters from the model for estimating the number of surviving fish under different scenarios. Concerning the first simulation experiment (a fixed number of fish is released at different ages/sizes), when fish are released at an age for which natural mortality is still very high (e.g., 500 days old), the number of surviving fish quickly decreases, mainly due to the disproportionate natural mortality. When fish are released at an age for which fishing mortality is high (e.g., 3,000 days old), the expected number of surviving fish also decreases, but in this case, it is related mainly to fishing mortality because natural mortality is small. However, at intermediate ages (e.g., 1,500 days old), both natural and fishing mortality are moderate and total mortality may reach a minimum (Fig 5).

**Fig 6:**
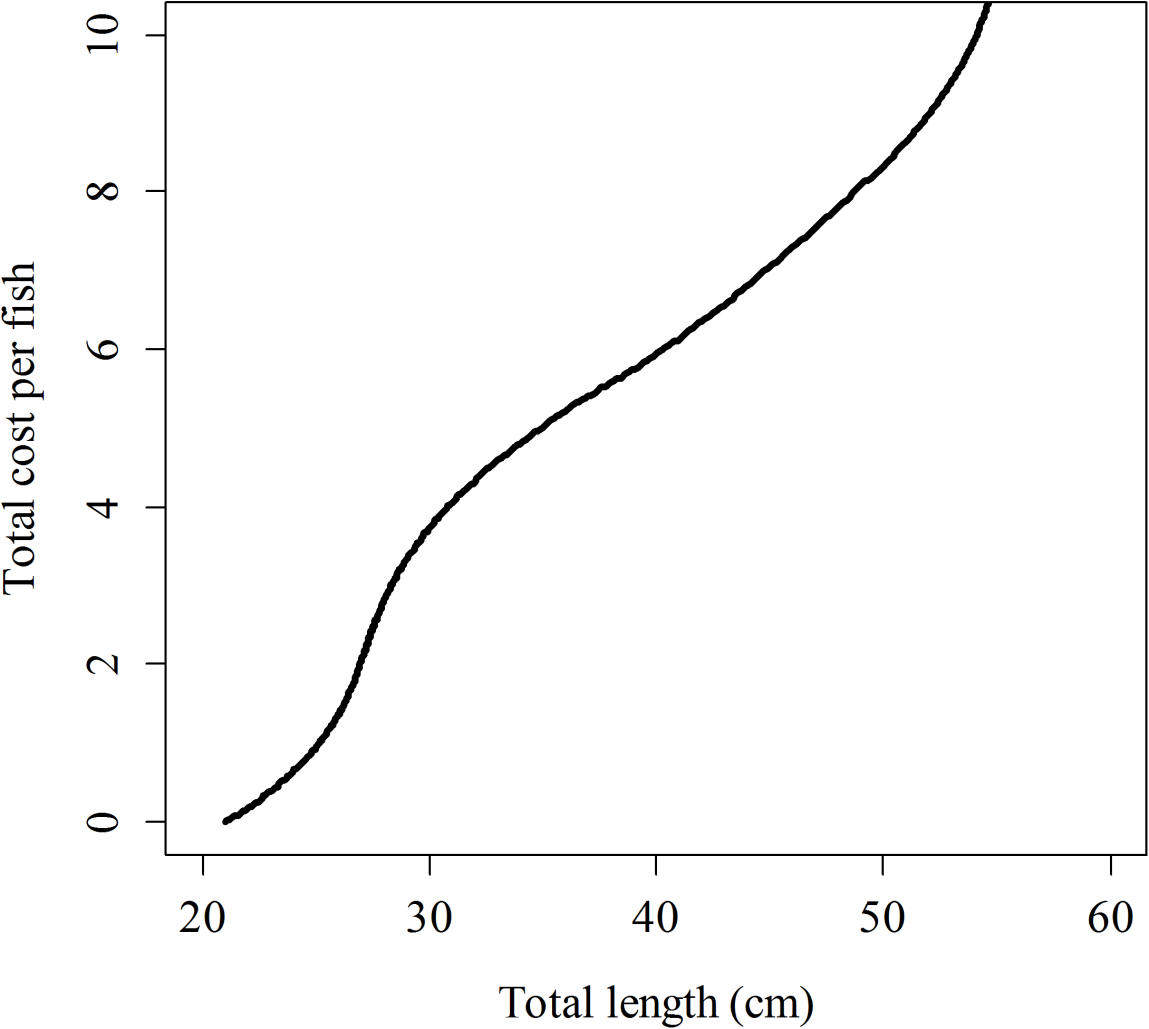
Size-dependent total production cost (€) for meagre juveniles kept in captivity conditions using the growing model and cost analysis developed by Gil, *et al*. [6].

**Fig 7:**
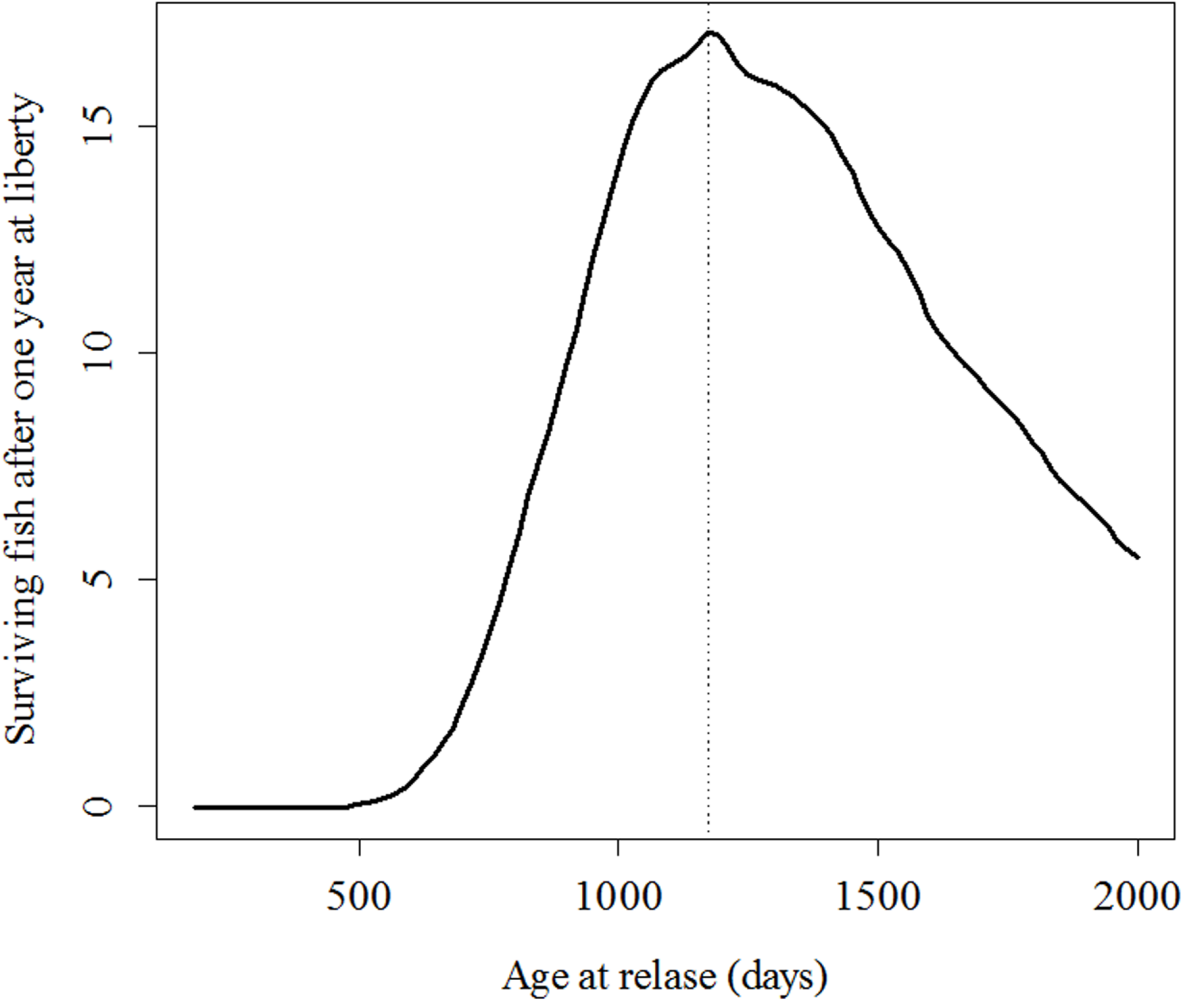
Expected number of surviving fish after one year at liberty based on the number of fish that can be produced with a fixed budget (10,000 €).

The second simulation experiment is very similar, but the number of fish actually released depends on a fixed budget (here, 10,000 €/year). The higher the length at the time of release, the smaller the number of fish actually produced is [6], because the cost of production per fish (*CO*) increase nonlinearly with the length and age (Fig 6). The releasing age that maximizes the number of survivors after one year at liberty was estimated at 1,173 days (Fig 7), but only 17 fish (out of the 201 fish that can be produced and released with 10,000 €/year) would survive. Therefore, in the case of meagres, there is no or very little chance that a large enough number of fish would be able to reach sexual maturity and recover the target population in a self-sustainable way, even when all fish are released at the optimal size/age. Nevertheless, the relevance of the method proposed here for optimizing the design of any other restocking program relies on the existence of an optimal release age and in the possibility of predicting the number of surviving fish in alternative scenarios. For example, just after reducing the adaptation period to *τ =* 100 days, the optimal release age reduces to only 310 days old; thus, a substantially larger number of fish can be produced and released with the same budget (3,752 fish), and from those released fish, up to 832 fish would survive after one year at liberty.

## Discussion

The aim of this study was to assess the success of the restocking program for the meagre in the Balearic Islands, but the experimental protocol proposed, the analytical strategy developed (Bayesian inference) and the results obtained have relevant implications for designing any other restocking program. The success of the program depends on how well and fast the hatchery-reared meagres, or any other released animals, can adapt to natural habitat conditions and survive. Griffith, *et al*. [33] reported that about one out of three restocking or reintroduction projects failed to create a self-sustaining population. This is because adaptation to the conditions of a new habitat is a task that many animals fail to accomplish [45, 46].

A total of 13,134 tagged and hatchery-reared meagres was released in Mallorca Island during the course of this restocking program. The pooled reported/released ratio was 3.3%, however a wide variability range was observed for the different release events. The reported/released ratio seems to be very variable even within the same restocking program, for example, from 0 to 31.3% for Atlantic cod [47] or from 2.6 to 23.3% for white sturgeon *Acipenser transmontanus* [17]. In our case, the largest meagres presented a higher reported/released ratio (14.1% and 23%). In contrast, ratios of 0% or close to 0% were observed when small fish were released. Clear evidence of the impact of the size-at-release on the reported/released ratio have been reported elsewhere [48], so the relevance of size-at-release should be considered well-established.

This fact may be due to the greater impact of predation on small organisms [49, 50] but also to the low resilience to starvation in small individuals or to their poor tolerance for environmental extremes [51–53]. Therefore, the mortality of released fish into the wild seems to be inversely correlated with fish size and age. Nevertheless, this relationship may be nonlinear, most likely because predation-related mortality would tend to stabilize after overcoming a critical size [48, 54].

An abnormally high mortality during the time just after release has been described by Hervas, *et al*. [14], who proposed that released fish have different survival for the short- and long-term. Short-term mortality has been attributed to the inexperience of recently released fish for both capturing prey and avoiding predators, and it appears to be high during a relatively short period [31, 55, 56]. For example, in the case of turbot, short-term mortality reaches 66% per day within the first three days after release [32], and Japanese flounder juveniles improved daily survival approximately 13 days after release [57].

These results seem to support the idea that natural mortality after release only (or mainly) depends on the time passed from release and in a lesser extent to age/size itself. Conversely, we propose a broader interpretation for the concept of adaptation period, and both short- and long-term mortality could be the outcome of the same biological processes: larger fish are more resilient to environmental stress and less vulnerable to predation; therefore, a larger percentage of large fish are able to overcome the critical period. Most of the small fish do not survive because of predation, environmental stress, or both. The model proposed here suggests that natural mortality varies between two values (i.e., initial mortality, *m*_*0*_, and final mortality, *m*_*∞*_), with the shift between them determined by the parameter *τ*. As natural mortality gradually decreases with fish size/age, the natural mortality just after release (*m*_*0*_) would be lower for larger fish. Furthermore, when released fish are able to adapt quickly to wild conditions, the natural mortality will decrease sharply. The meagre’s long adaptation period (*τ* = 298 days) would agree with the difficulties for shifting from a pellet-based diet to a wild diet (crustaceans and fish). Released meagres tend to show empty stomachs and bad body conditions during a long adaptation period [38]; therefore, many meagre experience a starvation period just after release, implying high mortality. Therefore, only large/old fish are able to overcome such severe environmental stresses.

Predation and starvation may play synergic roles in the case of the meagre. Although an analysis of potential meagre predators in the Balearic waters has not been conducted, significant populations of cormorants (*Phalacrocorax aristotelis*) have been reported, and they could be responsible for relevant mortality. Cormorants predate within sea cages at LIMIA facilities, but only on small meagres (unpublished data). Besides, in situations where mortality depends on spatial encounters between predators and fish, it has been predicted a smaller vulnerability of low activity phenotypes [58]. Therefore, it is possible that may exist some ontogenic change in activity of released fish. However, this statement has not been tested in this study.

Even so, some meagres were recaptured when they were 3–4 years old and, thus, were expected to be sexually mature. The age at maturity (*Age*_50_) was estimated to be 3.5 years for female meagres in the Balearic Islands [59]. Furthermore, these old recaptured females showed macroscopic and microscopic evidence of maturation in their gonads (unpublished data); therefore, they were potentially able to reproduce in the wild. In this specific sense, this restocking program could be considered successful. However, the estimated number of meagres that would be able to reach the maturity (9.6 specimens from the release #13) was very low and most likely too low to rebuild a well-structured broodstock and provide juveniles for the next generation.

Nevertheless, the relevance of the method proposed for optimizing the design of any other restocking program lies in the establishment of an optimal release age that would maximize the expected number of surviving fish. The optimal age (first simulation experiment) for releasing meagres was estimated to be approximately 1,500 days old. A smaller number of survivors are expected when releasing both younger and older fish. Natural mortality reduces with age, but this pattern may be counteracted by the opposite trend displayed by fishing mortality, which is size-dependent because most fishing gear is designed to catch larger and older fish [43, 44]. Such selectivity is economically driven: large fish are usually preferred by consumers and therefore have a higher market price. Most fishing gear is designed in a way that larger individuals have a higher chance of being caught [60]. In the case of the meagre, we propose that fishing mortality is zero for fish below 13.8 cm but linearly increases with fish size. Nevertheless, different relationships between size/age and fishing/hunting mortality can be considered for other animals and can be easily adapted to the statistical framework proposed here.

The production cost of juveniles may be even more important than the balance between fishing and natural mortality. Higher size-at-release implies more time at the facilities and higher production cost [6]. Provided that budgets are always limited, the number of fish that can be actually produced nonlinearly decreases with increasing age [6]. The optimal size at release would ultimately depend on all of these complex tradeoffs. In the case of the meagre, the optimal age of release was estimated to be 1,173 days old.

The proposed model strongly supports that the success of a restocking program would not be guaranteed by the release of more and more animals. Instead, reducing high post-release mortality is a key goal that needs further investigation. Optimizing the release strategies (such as modifying size-at-release, release habitat and microhabitat, release season and density) through pilot experiments can improve fish survival [50, 61, 62]. Furthermore, recent studies have demonstrated that conditioning reared fish before release could also improve behavioral skills such as feeding, burying and predator avoidance [63–66], resulting in higher survival of juveniles after release [32].

In conclusion, we emphasize the need to complete a pilot study consisting of multiple release events with between-event variability in the factors that may affect survival (here, size and age at release) to determine the optimum parameters for release. Tools for identifying the key processes that drive each specific system are provided in the analytical strategy proposed here. For example, the simulation experiments suggest that the number of fish surviving after one year can increase from 17 fish (current setting, i.e., *τ* = 298 days) to 832 fish after reducing *τ* to 100 days. Therefore, the method proposed would be a very useful tool for optimizing the design according to case-specific objectives and evaluating the success of any restocking or reintroduction program.

## Supporting Information

S1 File Sub-file A. R code implemented for the proposed model. Sub-file B. Data from the meagre restocking program used to evaluate the proposed model. Information from the 21 release events, such as number of released juveniles and their age and average size at release, and from the 413 meagre recaptures, such as the days spent at liberty and their release event, were provided for estimate the parameters of the model. (RAR)
